# Severity of Pneumonia in Under 5-Year-Old Children from Developing Countries: A Multicenter, Prospective, Observational Study

**DOI:** 10.4269/ajtmh.16-0733

**Published:** 2017-05-01

**Authors:** Thomas Bénet, Valentina Sanchez Picot, Shally Awasthi, Nitin Pandey, Ashish Bavdekar, Anand Kawade, Annick Robinson, Mala Rakoto-Andrianarivelo, Maryam Sylla, Souleymane Diallo, Graciela Russomando, Wilma Basualdo, Florence Komurian-Pradel, Hubert Endtz, Philippe Vanhems, Gláucia Paranhos-Baccalà, for the GABRIEL Network

**Affiliations:** 1 Laboratoire des Pathogènes Emergents, Fondation Mérieux, Centre International de Recherche en Infectiologie (CIRI), INSERM U1111, CNRS, UMR5308, ENS de Lyon, UCBL1, Lyon, France; 2 Service d'Hygiène, Epidémiologie et Prévention, Hôpital Edouard Herriot, Hospices Civils de Lyon, Lyon, France; 3 Chhatrapati Shahu Ji Maharaj University, Lucknow, India;; 4 KEM Hospital Research Centre, Pune, India; 5 Hôpital Femme-Mère-Enfant, Antananarivo, Madagascar; 6 Fondation Mérieux, Centre d'Infectiologie Charles Mérieux (CICM), Antananarivo, Madagascar; 7 Gabriel Touré Hospital, Bamako, Mali; 8 Centre d'Infectiologie Charles Mérieux (CICM), Bamako, Mali; 9 Health Research Institute, Asuncion, Paraguay; 10 Hospital Pediátrico “Niños de Acosta Ñu,” San Lorenzo, Paraguay; 11 Department of Medical Microbiology and Infectious Diseases, Erasmus MC, Rotterdam, The Netherlands

## Abstract

Pneumonia is the leading cause of death in children. The objectives were to evaluate the microbiological agents linked with hypoxemia in hospitalized children with pneumonia from developing countries, to identify predictors of hypoxemia, and to characterize factors associated with in-hospital mortality. A multicenter, observational study was conducted in five hospitals, from India (Lucknow, Vadu), Madagascar (Antananarivo), Mali (Bamako), and Paraguay (San Lorenzo). Children aged 2–60 months with radiologically confirmed pneumonia were enrolled prospectively. Respiratory and whole blood specimens were collected, identifying viruses and bacteria by real-time multiplex polymerase chain reaction (PCR). Microbiological agents linked with hypoxemia at admission (oxygen saturation < 90%) were analyzed by multivariate logistic regression, and factors associated with 14-day in-hospital mortality were assessed by bivariate Cox regression. Overall, 405 pneumonia cases (3,338 hospitalization days) were analyzed; 13 patients died within 14 days of hospitalization. Hypoxemia prevalence was 17.3%. Detection of human metapneumovirus (hMPV) and respiratory syncytial virus (RSV) in respiratory samples was independently associated with increased risk of hypoxemia (adjusted odds ratio [aOR] = 2.4, 95% confidence interval [95% CI] = 1.0–5.8 and aOR = 2.5, 95% CI = 1.1–5.3, respectively). Lower chest indrawing and cyanosis were predictive of hypoxemia (positive likelihood ratios = 2.3 and 2.4, respectively). Predictors of death were *Streptococcus pneumoniae* detection by blood PCR (crude hazard ratio [cHR] = 4.6, 95% CI = 1.5–14.0), procalcitonin ≥ 50 ng/mL (cHR = 22.4, 95% CI = 7.3–68.5) and hypoxemia (cHR = 4.8, 95% CI = 1.6–14.4). These findings were consistent on bivariate analysis. hMPV and RSV in respiratory samples were linked with hypoxemia, and *S. pneumoniae* in blood was associated with increased risk of death among hospitalized children with pneumonia in developing countries.

## Introduction

Despite reduced mortality rates in recent years, pneumonia is the foremost cause of death from infectious diseases in under 5-year-old children worldwide, accounting for 15% of total deaths, mostly in developing countries.^1^ Hypoxemia, frequently associated with pneumonia and a marker of disease severity, leads to 3- to 4-fold increased risk of death in children with pneumonia.^2^ A Cochrane review^3^ has reported that systematic hypoxemia screening with pulse oximetry and appropriate oxygen supply are effective in preventing death from pneumonia in children.

A large simulation study estimated that systematic pulse oximetry may globally prevent almost 150,000 deaths from pneumonia annually.^4^ However, the microbiological agents linked with hypoxemic pneumonia are poorly recognized. The identification of such etiological agents would serve to better target preventive (i.e., vaccination) and curative measures (i.e., antibiotics and antiviral drugs), reducing the global burden of hypoxemia and pneumonia. Because of its high incidence and related mortality, particular attention must be paid to hypoxemic pneumonia in developing countries. Pulse oximetry is still rarely available in health-care settings of developing countries.^5^ In the absence of oximeter, hypoxemia can be detected by several clinical signs or symptoms, including cyanosis and increased respiratory rate. However, none is sensitive and specific enough to reliably detect hypoxemia.^6^^–^^8^ Most studies of hypoxemia in children with pneumonia were performed in one country and rarely investigated the relationship between hypoxemia and microbiological results, so it would be useful to reassess them in a more recent multicontinental investigation.^2^

The risk factors of death among children with pneumonia in developing countries have already been identified,^9^^,^^10^ but rarely regarding the relationship between microbiological findings and mortality. Assessment of clinical, para-clinical, and microbiological predictors of death would be useful to prioritize public health campaigns. Identification of microbiological agents associated with death and/or hypoxemia would be useful to better focus therapeutic measures. Indeed, hypoxemic pneumonia can be treated with oxygen in conjunction with other measures, whereas non-hypoxemic pneumonia with poor vital prognosis might need other regimens, such as antibiotics/antivirals or intensive care.

The objectives of the present study are to assess the microbiological agents linked to hypoxemia in hospitalized children with pneumonia in developing countries, to identify clinical and para-clinical predictors of hypoxemia and to pinpoint factors associated with death within 2 weeks after admission.

## Materials and Methods

### Study sites and design.

Findings from a prospective, hospital-based, multicenter, longitudinal study, conducted at five sites in four countries located on three continents, were analyzed: Lucknow and Vadu in India, Antananarivo in Madagascar, Bamako in Mali, and San Lorenzo in Paraguay. The participating sites are members of the GABRIEL (Global Approach to Biological Research, Infectious Diseases and Epidemics in Low-Income Countries) Network established by Fondation Mérieux.^11^ The study protocol and sites are described elsewhere.^12^ Pneumonia cases from the original case–control study were followed up during hospital stay and constituted the analyzed cohort.

The study population comprised children aged between 2 and 60 months, complying with protocol definitions and inclusion criteria. Eligible patients were identified by study clinicians at each participating site. All consecutive patients hospitalized in pediatric departments, who were eligible for study entry, were enrolled during each season (dry and rainy) for at least a 1-year period. The study aimed at obtaining an equal number of individuals in each season at each study site. Incident cases were defined as hospitalized children aged 2–60 months, with clinical features of pneumonia, as described latter, radiological confirmation of pneumonia on chest X-ray as per World Health Organization (WHO) guidelines,^13^ and informed consent statement signed by the children's parents or legal guardian. Wheezing at auscultation was initially an exclusion criterion, but was amended because it slowed the inclusion process. Thus, it was finally decided to include children presenting pneumonia with or without “wheezing.”

The present study selectively comprised sites with better quality data on oxygen saturation (SO_2_) at admission, mortality among pneumonia cases, and documented recording of patient follow-up during hospitalization. Main characteristics were compared by site, and multivariate analysis accounted for heterogeneity of sites regarding observed or non-observed potential confounding factors. Patients with missing data on follow-up, SO_2_ measurement, and vital status at discharge were excluded (*N* = 59). Excluded patients did not differ from those analyzed for gender and weight-for-height *Z* score, but were older (*P* = 0.03).

### Definition of pneumonia.

Pneumonia cases were defined by the following criteria:

• Cough and/or dyspnea.
• Tachypnea, as delineated by the WHO (in children 2–12 months of age: breathing rate ≥ 50 cycles per minute; in children 12–59 months of age: breathing rate ≥ 40 cycles per minute).^14^
• First symptoms appearing within the last 14 days.
• Radiological confirmation of pneumonia as per WHO guidelines, including primary endpoint pneumonia or other infiltrates.^13^

### Data sources and quality control.

Data quality was monitored and evaluated by each site and by the Emerging Pathogens Laboratory (Lyon, France) for pooled data analysis. Demographic characteristics, underlying diseases, medical history, clinical examination at enrollment, therapeutics, vaccinations, and outcomes were recorded prospectively for each patient on a standardized paper form. Each potential error was discussed with local investigators, and a final ruling was applied. The principal investigator at each site was informed about quality assessments and was involved in their resolution. Hypoxemia was defined as SO_2_ < 90%, according to WHO recommendations.^15^ SO_2_ was measured at hospital admission, before the administration of oxygen or other therapeutics. Vital status was recorded until patient discharge.

### Biological samples.

Samples were collected in the first 48 hours of patient hospitalization. Nasal swabs/aspirates, whole blood, and pleural effusions (in case of pleurisy) were sampled from all patients. Urine was collected at patient admission to ascertain history of antibiotic use. Biological samples were taken before the in-hospital administration of antibiotics. Whole blood allowed complete blood count and culture, with real-time multiplex polymerase chain reaction (RT-PCR) assay for the identification of *Staphylococcus aureus*, *Streptococcus pneumoniae*, and *Haemophilus influenzae* type B. C reactive protein (CRP) and procalcitonin (PCT) were quantified in serum. Respiratory specimens permitted the identification of viruses and bacteria by RT-PCR assay with a panel of 19 viruses and five bacteria (Fast-track Diagnostic respiratory pathogens 21 plus, Fast-track diagnostic, Esch-sur-Alzette, Luxemburg), namely: influenza virus A, influenza virus A/H1N1, influenza virus B, coronavirus 229E, coronavirus OC43, coronavirus NL63, coronavirus HKU1, human parainfluenza virus 1, 2, 3, and 4, human metapneumoviruses (hMPV) A and B, rhinovirus, respiratory syncytial virus (RSV) A and B, adenovirus, enterovirus, parechovirus, bocavirus, *Mycoplasma pneumoniae*, *Chlamydia pneumoniae*, *S. aureus*, *S. pneumoniae*, and *H. influenzae* type B. *Streptococcus pneumoniae*-positive specimens were serotyped by multiplex RT-PCR that detects 29 different serotypes. A centralized, blinded PCR respiratory quality control panel was provided to all sites to ensure procedure validation on-site before specimens were processed locally.

### Statistical methods.

Qualitative variables were described as numbers and percentages with comparison by χ^2^ test, if appropriate, or Fischer's exact test. Quantitative variables were reported as median and interquartile range (IQR) and compared by the Mann–Whitney *U* test or Kruskal–Wallis one-way analysis of variance. Positive likelihood (LR+) and negative likelihood (LR−) ratios of various clinical signs and symptoms were calculated to detect hypoxemic cases with the following formulae: LR+ = sensitivity/(1 − specificity) and LR− = (1 − sensitivity)/specificity. It has been underlined that LR calculation is useful to improve diagnostic accuracy.^16^ LR could thus express the proportion of hypoxemic children who presented a particular sign or symptom divided by the proportion of non-hypoxemic children with the same result.

Microbiological findings from different sample sites associated with hypoxemia were assessed by logistic regression modeling. Multivariate analysis was performed after univariate analysis, with forced adjustment on patient age, time per quarter, and study center. Microorganisms with *P* < 0.15 values on univariate analysis were initially entered in the multivariate model. Thus, backward stepwise deletion was applied until all *P* values were < 0.05. Models were compared by Wald testing.

Factors associated with in-hospital mortality were assessed with Kaplan–Meier curves and compared by log-rank test. Follow-up was censored at 14 days after admission or discharge, if duration of hospitalization was less than 14 days. The characteristics of patients deceased within 2 weeks (*N* = 13) were compared with non-deceased patients (*N* = 392). Univariate and bivariate proportional hazard Cox regression analyses were undertaken. No multivariate Cox model was fitted owing to the limited number of events. Bivariate analyses expressed the effect of one major risk factor from univariate analysis adjusted on one other possible confounder (age category, human immunodeficiency virus [HIV] seropositivity, time per quarter, or weight-for-height *Z* score). All tests were two tailed, and *P* < 0.05 was considered significant. Statistical analysis was conducted with Stata version 13.0 (StataCorp., College Station, TX).

### Ethics.

The study protocol, informed consent statement, clinical research form, amendments, and all other study documents were submitted to and approved by the institutional research ethics committee of each site.

## Results

### Population description.

Overall, 405 children with pneumonia, accounting for 3,338 hospitalization days, were included. Among them, 235 (58.0%) were male. Median age was 14 months (IQR = 8–27 months). Ninety-six (23.7%) patients came from Lucknow, India, 71 (17.5%) were from Vadu, India, 26 (6.4%) were from Antananarivo, Madagascar, 114 (28.1%) from Bamako, Mali, and 98 (24.2%) from San Lorenzo, Paraguay. The study periods at each site were August 7, 2012, to December 6, 2013, in Lucknow; June 23, 2012, to March 5, 2014, in Vadu; February 4, 2011, to December 13, 2012, in Antananarivo; July 4, 2011, to November 14, 2012, in Bamako; and May 18, 2010, to May 20, 2013, in San Lorenzo.

Patients differed between sites according to median weight-for-height *Z* score (*P* = 0.001) and age category (*P* = 0.007), but did not differ between sites according to HIV seropositivity (*P* = 0.33) and mortality (*P* = 0.37). Seventy patients were hypoxemic at admission. Global prevalence of hypoxemia was 17.3% (95% confidence interval [CI] = 13.9–21.3%). Median SO_2_ was 95% (IQR = 92–97%), without differences between countries (from 95% [IQR = 87–96%] in Mali to 96% [IQR = 89–98%] in Vadu, India, *P* = 0.14). Table 1

**Table 1:** Description of hypoxemic and non-hypoxemic pneumonia cases (*N* = 405)

Characteristics at admission	Hypoxemic* pneumonia (*N* = 70)	Non-hypoxemic* pneumonia (*N* = 335)	*P*
Demographics			
Gender, male	38/70 (54.3)†	197/335 (58.8)†	0.49
Age, months, median (IQR)	12 (4–23)	15 (8–30)	0.02
Age category			0.13
2–11 months	32/70 (45.7)	132/335 (39.4)	
12–23 months	21/70 (30.0)	80/335 (23.9)	
24–60 months	17/70 (24.3)	123/335 (36.7)	
Weight-for-height *Z* score, median (IQR)	−1.4 (−2.9; 0)	−1.1 (−2.1; 0.3)	0.20
Weight-for-height *Z* score ≤ 2 SD	27/67 (40.3)	65/233 (27.9)	0.05
Weight-for-height *Z* score ≤ 3 SD	14/67 (20.9)	31/233 (13.3)	0.12
Delay from onset, days, median (IQR)	6 (3–7)	5 (3.5–7)	0.07
Medical history			
Heart disease	6/69 (8.7)	21/335 (6.3)	0.46
Lung disease	4/67 (6.0)	32/335 (9.5)	0.35
Asthma	0/69 (0)	3/335 (0.9)	0.43
HIV positive	1/61 (1.6)	2/239 (0.8)	0.57
Contracted common cold/pharyngitis‡	34/69 (49.3)	86/330 (26.1)	< 0.001
Previous tuberculosis	0/68 (0)	2/335 (0.6)	0.52
Contact with a tuberculosis case	0/68 (0)	1/298 (0.3)	0.63
Prior treatment of fever	44/70 (62.9)	255/335 (76.1)	0.02
Pneumococcal conjugate vaccine	2/54 (3.7)	15/297 (5.0)	0.67
DPT-HepB-Hib vaccine, one dose	59/67 (88.1)	191/303 (63.0)	< 0.001
DPT-HepB-Hib vaccine, three doses	45/65 (69.2)	156/291 (53.6)	0.02
Vital signs at admission			
Temperature, °C, median (IQR)	38.4 (37.5–39)	38.3 (37.8–38.8)	0.50
Breathing rate, cycles/minute, median (IQR)	58 (54–68)	56 (49–64)	0.009
Cardiac rate, cycles/minute, median (IQR)	151 (136–167)	140 (123–156)	0.001
Systolic pressure, mmHg, median (IQR)	90 (80–92)	92 (88–100)	0.007
Diastolic pressure, mmHg, median (IQR)	60 (50–61)	66 (60–70)	0.002
SO_2_, %, median (IQR)	85 (80–87)	96 (94–97)	< 0.001
Clinical signs/symptoms at admission			
Dyspnea	67/70 (95.7)	324/335 (96.7)	0.68
Lower chest indrawing	63/70 (90.0)	257/333 (77.2)	0.02
Cough	67/68 (98.5)	330/335 (98.5)	0.99
Pulmonary crackles	62/70 (88.6)	301/334 (90.1)	0.70
Rhonchi	14/62 (22.6)	38/245 (15.5)	0.18
Wheezing	5/70 (7.1)	50/332 (15.1)	0.08
Rhinopharyngitis	11/69 (15.9)	61/334 (18.3)	0.65
Prostration or lethargy	29/69 (42.0)	109/335 (32.5)	0.13
Inability to drink	14/69 (20.3)	54/334 (16.2)	0.40
Diarrhea	4/70 (5.7)	44/335 (13.1)	0.08
Cyanosis	10/70 (14.3)	16/333 (4.8)	0.003
Vomiting	7/69 (10.1)	49/334 (14.7)	0.32
Convulsions	3/69 (4.3)	13/335 (3.9)	0.86
Conjunctivitis	4/69 (5.8)	4/335 (1.2)	0.01
Diminished breathing sounds	25/61 (41.0)	90/244 (36.9)	0.55
Dullness to percussion	18/70 (25.7)	84/332 (25.3)	0.94
Otitis	0/70 (0)	3/334 (0.9)	0.43
Rasping	4/62 (6.4)	42/245 (17.1)	0.03
Radiology			
Generalized, dense, homogenous opacification	30/69 (43.5)	92/334 (27.5)	0.009
Other findings	39/69 (56.5)	242/334 (72.5)	
Pleural effusion	5 (7.3)	78 (23.4)	0.003
Biology at admission			
White blood cell count, ×10^9^ cells/L, median (IQR)	22 (8.9–29.1)	11.5 (1–23.9)	< 0.001
White blood cell count > 20 × 10^9^ cells/L	37/67 (55.2)	71/235 (30.2)	< 0.001
Neutrophils, %, median (IQR)	47 (27–68)	45 (28–64)	0.70
C reactive protein, mg/L, median (IQR)	18 (8–63)	24 (6–94)	0.58
Procalcitonin, ng/mL, median (IQR)	4.3 (0.4–16.2)	1.6 (0.2–6.6)	0.03
Procalcitonin > 50 ng/mL	10/62 (16.3)	16/306 (5.2)	0.002
Positive antibiotic urinary test	35/42 (83.3)	187/210 (89.0)	0.30
During hospital stay			
Oxygen	64/69 (92.7)	196/333 (58.9)	< 0.001
Oxygen duration, days, median (IQR)	2 (1–3)	2 (1–3)	0.39
Antibiotics	62/63 (98.4)	295/296 (99.7)	0.23
Antibiotics duration, days, median (IQR)	6 (4–8)	7 (5–10)	0.02
Other therapy	64/68 (94.1)	220/317 (69.4)	< 0.001
Hospitalization length, days, median (IQR)	5.5 (4–9)	7 (4–11)	0.03
Recovery	58/68 (85.3)†	314/334 (94.0)†	0.01
Death	6/70 (8.6)	8/335 (2.4)	0.01
Transfer	3/69 (4.3)	4/335 (1.2)	0.07

DPT-HepB-Hib = diphtheria, pertussis, tetanus, hepatitis B, and *Haemophilus influenzae* type b; HIV = human immunodeficiency virus; IQR = interquartile range; SD = standard deviation; ILI = influenza-like illness; SO_2_ = oxygen saturation.

* SO_2_ lower than 90%.

† Expressed as number/number with available data (%), unless specified otherwise.

‡ Within 2 weeks.

compares the characteristics of hypoxemic and non-hypoxemic patients.

HIV prevalence was 1.0% (*N* = 3). Median weight-for-height *Z* score was −1.1 (IQR = −2.4; +0.1). Median length of hospital stay was 7 days (IQR = 4–10 days). Median CRP level at admission was 24 mg/L (IQR = 6–90 mg/L), median white blood cell count was 12,600 × 10^9^ cells/L (IQR = 1,000–25,000 × 10^9^ cells/L), and median neutrophil proportion was 47% (IQR = 28–64%). Median PCT level at admission was 1.9 ng/mL (IQR = 0.3–8.1 ng/mL), with mean of 15.6 ng/mL (minimum: 0.05, maximum: 585.5 ng/mL).

Overall, 70.5% tested positive for urinary antibiotics at admission. Among the 402 (99.3%) patients given antibiotics during hospitalization for a median duration of 7 days (IQR = 5–10 days), 298 (74.5%) received monotherapy, with some also getting multiple antibiotic lines. The main drugs were ceftriaxone (*N* = 135, 26.0%), amoxicillin (*N* = 120, 23.1%), ampicillin (*N* = 51, 9.8%), amoxicillin/sulbactam (*N* = 36, 6.9%), amoxicillin/clavulanic acid (*N* = 35, 6.7%), oxacillin (*N* = 35, 6.7%), and vancomycin (*N* = 22, 4.2%).

### Microbiological agents associated with hypoxemia in children with pneumonia.

Hypoxemic (*N* = 70) and non-hypoxemic (*N* = 335) pneumonia cases did not differ by median number of bacteria (1 versus 1, respectively, *P* = 0.85) or viruses detected (1 versus 1, respectively, *P* = 0.72) in nasal swabs/aspirates. Infection types (bacterial/viral/mixed) did not differ in hypoxemic and non-hypoxemic patients (*P* = 0.96). Table 2

**Table 2:** Microbiological agents associated with hypoxemia in children with pneumonia (*N* = 405)

Microbiological agent	Hypoxemic* pneumonia (*N* = 70)	Non-hypoxemic* pneumonia (*N* = 335)	*P*	Crude odds ratio (95% CI)	Adjusted odds ratio† (95% CI)
Respiratory sampling					
*Streptococcus pneumoniae*	44/70 (62.9)‡	202/335 (60.3)‡	0.69	1.1 (0.7–1.9)	–
*Staphylococcus aureus*	12/69 (17.4)	58/335 (17.3)	0.99	1.0 (0.5–2.0)	–
*Haemophilus influenzae*	4/70 (5.7)	17/335 (5.1)	0.83	1.1 (0.4–3.5)	–
*Mycoplasma pneumoniae*	0/70 (0)	3/335 (0.9)	0.43	NE	–
*Chlamydia* spp.	0/70 (0)	1/335 (0.4)	0.65	NE	–
hMPV	10/70 (14.3)	23/335 (6.9)	0.04	2.3 (1.0–5.0)	2.4 (1.0–5.8)
Coronavirus 63	1/70 (1.4)	1/335 (0.3)	0.22	4.8 (0.3–78.3)	–
Coronavirus 229	1/70 (1.4)	3/335 (0.9)	0.68	1.6 (0.2–15.6)	–
Coronavirus 43	1/70 (1.4)	14/335 (4.2)	0.27	0.3 (0.04–2.6)	–
HKU	2/70 (2.9)	9/335 (2.7)	0.94	1.1 (0.2–5.0)	–
Adenovirus	4/70 (5.7)	23/335 (7.8)	0.55	0.7 (0.2–2.1)	–
Enterovirus	4/70 (5.7)	22/335 (6.6)	0.79	0.9 (0.3–2.6)	–
Parechovirus	1/37 (2.7)	1/254 (0.11)	0.11	4.8 (0.3–78.3)	–
Rhinovirus	17/70 (24.3)	97/335 (29.0)	0.43	0.8 (0.4–1.4)	–
RSV	18/70 (25.7)	44/335 (13.1)	0.008	2.3 (1.2–4.3)	2.5 (1.1–5.3)
hPIV 1	3/70 (4.3)	13/335 (3.9)	0.87	1.1 (0.3–4.0)	–
hPIV 2	1/70 (1.4)	1/335 (0.3)	0.22	4.8 (0.3–78.3)	–
hPIV 3	1/70 (1.4)	21/335 (6.3)	0.10	0.2 (0.03–1.6)	–
hPIV 4	2/70 (2.9)	10/335 (3.0)	0.95	1.0 (0.2–4.5)	–
Influenza virus A	4/70 (5.7)	24/335 (7.2)	0.66	0.8 (0.3–2.3)	–
Influenza virus B	0/70 (0)	8/335 (2.4)	0.19	NE	–
Influenza virus A H1/N1	2/70 (2.9)	9/335 (2.7)	0.94	1.1 (0.2–5.0)	–
Bocavirus	3/70 (4.3)	23/335 (6.9)	0.42	0.6 (0.2–2.1)	–
Blood sample					
*Streptococcus pneumoniae*	10/70 (14.3)	41/335 (12.2)	0.64	1.2 (0.6–2.5)	–
*Staphylococcus aureus*	3/70 (4.3)	5/335 (1.5)	0.13	3.0 (0.7–12.7)	–
*Haemophilus influenzae*	3/70 (4.3)	15/335 (4.5)	0.94	1.0 (0.3–3.4)	–

CI = confidence interval; hMPV = human metapneumovirus; hPIV = human parainfluenza virus; NE = non-estimable; RSV = respiratory syncytial virus.

* SO_2_ lower than 90%.

† After multivariate logistic regression, adjusted on other microorganisms significantly associated with hypoxemia, patient age, time period per quarter and center.

‡ Data are expressed as number positive/number with available data with % unless specified otherwise.

**Figure 1.**
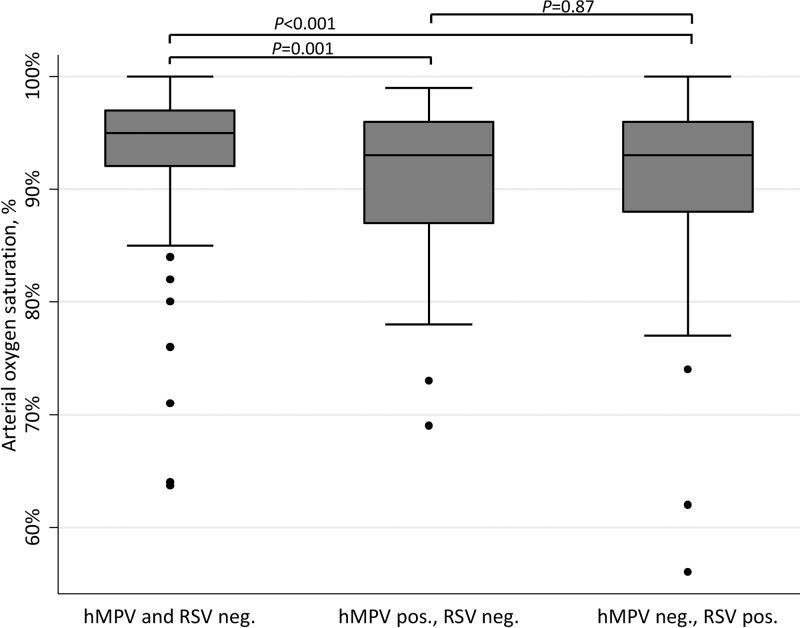
Oxygen saturation (SO_2_) in children with RSV or hMPV pneumonia, *N* = 404. Mean SO_2_ was 93.9% (+5.0) in hMPV- and RSV-negative patients (*N* = 311), 90.7% (+8.1) in hMPV-positive patients (*N* = 32), and 90.4% (+8.5) in RSV-positive patients (*N* = 61). One patient coinfected by hMPV and RSV was excluded from this analysis: his SO_2_ was 96%. SO_2_ between groups was compared by Student's *t* test. hMPV = human metapneumovirus; neg. = negative; RSV = respiratory syncytial virus; pos. = positive.

reports the microbiological agents linked with hypoxemia. Univariate analysis disclosed that hMPV and RSV detection in nasal samples was associated with increased risk of hypoxemia (*P* = 0.04 and 0.008, respectively). After adjustment on age, center, and calendar time, microorganisms independently associated with heightened risk of hypoxemia were hMPV (adjusted odds ratio [aOR] = 2.5, 95% CI = 1.1–5.3) and RSV (aOR = 2.4, 95% CI = 1.0–5.8). Median SO_2_ was lower in RSV- and hMPV-positive patients than in -negative patients but not different between RSV- and hMPV-positive patients (Figure 1

). Pneumococcus serotypes 6AB and 10A from respiratory samples were more frequent in hypoxemic than in non-hypoxemic patients (22.9% versus 9.5%, respectively, *P* = 0.002; 7.1% versus 1.5%, respectively, *P* = 0.006). The distribution of other serotypes was not significantly different in hypoxemic and non-hypoxemic children (Supplemental Figure 1).

### Clinical and para-clinical presentation in children with hypoxemic pneumonia.

Hypoxemic patients differed from non-hypoxemic patients according to age (*P* = 0.02), history of common cold/pharyngitis (*P* < 0.001), receipt of one dose of pentavalent vaccine (*P* < 0.001), breathing rate (*P* = 0.009), blood pressure (*P* < 0.01), chest indrawing (*P* = 0.02), cyanosis (*P* = 0.003), conjunctivitis (*P* = 0.01), rasping (*P* = 0.03), radiological presentation (*P* = 0.009), mean white blood cell count (*P* < 0.001), and PCT (*P* < 0.001) at admission (Table 1). LR+ of lower chest indrawing was 2.3 (95% CI = 1.1–4.9), and LR- was 0.9 (95% CI = 0.8–0.95). LR+ of cyanosis was 2.4 (95% CI = 1.4–4.1) and LR− was 0.7 (95% CI = 0.5–0.99). Other signs and symptoms were less predictive of hypoxemia (data not shown). Hypoxemic patients differed from non-hypoxemic patients in mean white blood cell count (*P* = 0.001) and PCT at admission (*P* = 0.03) but not regarding CRP level.

### Factors associated with death.

Fourteen (3.5%) patients died during hospital stay. Among them, 13 died within 14 days after hospital admission. The mortality rate was 8.6% in hypoxemic and 2.4% in non-hypoxemic patients (*P* = 0.01). Eight deceased patients were not hypoxemic at admission. Among them, the causes of death were multiple organ dysfunction syndrome (*N* = 4), acute respiratory distress syndrome with septic shock (*N* = 2), severe pneumonia in HIV (*N* = 1), and cardiac arrest (*N* = 1).

Table 3

**Table 3:** Factors associated with death of pneumonia in children within 2 weeks after hospital admission

Characteristics at admission	Deceased (*N* = 13)	Alive (*N* = 392)	*P*	Crude hazard ratio (95% CI)
*Streptococcus pneumoniae*, PCR blood	5/13 (38.5)*	46/392 (11.7)*	0.01	4.6 (1.5–14.0)
hPIV 2, nasal swab/aspirate	1/13 (7.7)	1/392 (0.3)	0.06	23.6 (3.0–183.9)
Hypoxemia	6/13 (46.1)	64/392 (16.3)	0.01	4.8 (1.6–14.4)
HIV positive	1/10 (10.0)	2/290 (0.7)	0.10	9.6 (1.2–75.9)
Weight-for-height *Z* score, median (IQR)	−2.1 (−4.3, −0.6)	−1.1 (−2.3, −0.1)	0.05	1.01 (0.99–1.04)
Weight-for-height *Z* score ≤ 3 SD	4/11 (36.4)	41/289 (14.2)	0.06	2.8 (0.8–9.5)
Inability to drink	5/13 (38.5)	63/390 (16.1)	0.05	2.7 (0.9–8.4)
Procalcitonin, ng/mL, median (IQR)	71.5 (8.3–111.5)	1.6 (0.3–6.6)	< 0.001	1.11 (1.07–1.15)†
Procalcitonin ≥ 50 ng/mL	8/13 (61.5)	18/355 (5.1)	< 0.001	22.4 (7.3–68.5)
Oxygen saturation, %, median (IQR)	91 (86–96)	95 (92–97)	0.04	2.1 (1.3–3.5)‡

CI = confidence interval; HIV = human immunodeficiency virus; hPIV = human parainfluenza virus; IQR = interquartile range; SD = standard deviation.

* Expressed as number/number with available data (%) unless specified otherwise.

† Per 10 ng/mL increase.

‡ Per 10% decrease.

**Figure 2.**
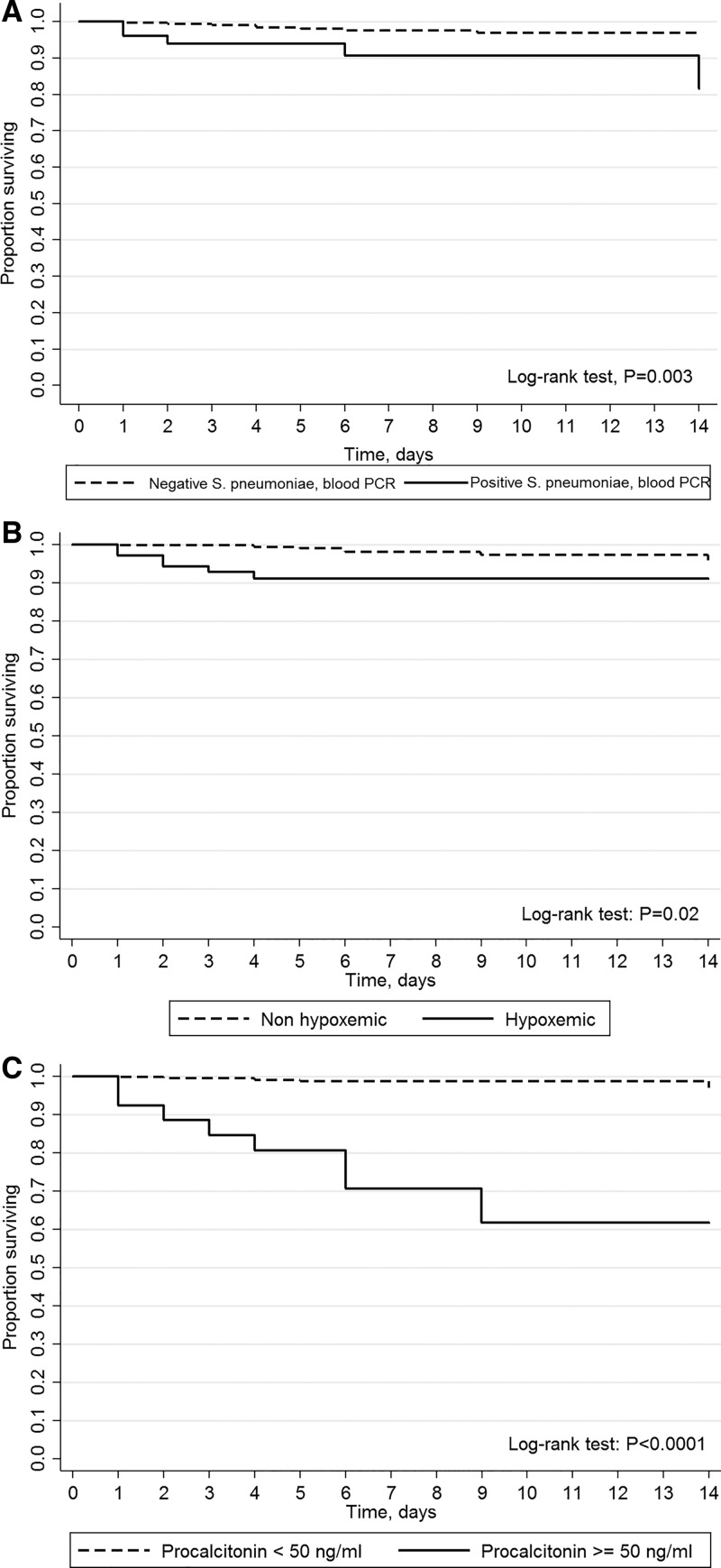
Kaplan–Meier curves of in-hospital survival of patients with pneumonia, *N* = 405. (**A**) *Streptococcus pneumoniae* positive vs. negative on blood PCR. (**B**) Hypoxemic (SO_2_ < 90%) vs. non-hypoxemic patients. (**C**) Procalcitonin > 50 vs. < 50 ng/mL. Time 0 was day of hospital admission. Follow-up was censored at patient discharge, death or 14 days after admission if duration of hospitalization was longer.

reports the microbiological, clinical, and para-clinical findings associated with death on univariate Cox analysis. *Streptococcus pneumoniae* detection by blood PCR, hypoxemia, and PCT ≥ 50 ng/mL at admission were associated with increased risk of death (log-rank test: *P* = 0.003, *P* = 0.002, and *P* < 0.0001, respectively, Figure 2A–C

). Other characteristics or microorganisms, including *S. pneumoniae* in respiratory samples, were not significantly associated with death (Supplemental Tables 1 and 2). No *S. pneumoniae* serotype was significantly associated with higher mortality (data not shown). *Streptococcus pneumoniae* detection by blood PCR, hypoxemia and PCT ≥ 50 ng/mL at admission were linked with augmented probability of death on univariate survival analysis (crude hazard ratio [cHR] = 4.6, 95% CI = 1.5–14.0; cHR = 4.8, 95% CI = 1.6–14.4; cHR = 22.4, 95% CI = 7.3–68.5, respectively). These associations were consistent on bivariate Cox regression with adjustment on country, age category, HIV seropositivity, time (per quarter), or weight-for-height *Z* score (Supplemental Figure 2). In addition, *S. pneumoniae* detection by blood PCR was associated with increased risk of death (aOR = 4.0, 95% CI = 1.3–12.4), independently of hypoxemia at admission (aOR = 4.3, 95% CI = 1.4–12.8).

## Discussion

One of the objectives of this study was to assess microbiological agents and other predictors of hypoxemia and death in under 5-year-old hospitalized children with pneumonia from developing countries. We observed that two viruses, namely RSV and hMPV, detected in respiratory samples by PCR, were independently associated with increased risk of hypoxemia, while no bacterial agent was significantly linked with it. On the other hand, *S. pneumoniae* detection by blood PCR was associated with a higher rate of in-hospital mortality in the study population independently of hypoxemia at admission. Several predictors of hypoxemic pneumonia were identified. However, none had high likelihood ratio. Elevated PCT concentration and hypoxemia were straightforward predictors of death in children with pneumonia. The contribution of hypoxemia to the risk of death was independent of pneumococcus detection by blood PCR.

Several studies have investigated factors associated with hypoxemia, particularly clinical predictors.^6^^–^^8^^,^^17^ However, few of them have researched the links between microbiological findings and severity in different developing countries, with a standardized protocol. We observed that two viruses were associated with increased risk of hypoxemia. Viral pneumonia induced diffuse, bilateral, pulmonary damage,^18^ compared with bacterial pneumonia, with more frequent, well-systematized alveolar localization.^19^ This is probably the reason why we noted that the two viral etiological agents evoking pneumonia were associated with hypoxemia.

RSV is the leading cause of viral pneumonia in children,^20^ frequently in association with severe disease.^21^ We observed that it might be also a major cause of hypoxemic pneumonia. On the other hand, RSV detection was not related to increased mortality, but it is estimated that 66,000–199,000 children could die of RSV-associated pneumonia worldwide every year. Our study's power was probably too limited to demonstrate such associations.

However, with prevalence of exposure in non-hypoxemic patients ranging from 10% to 50%, with bilateral tests and α < 0.05, study power was ≥ 80% to detect OR ≥ 2.5. Similar results have been reported recently in a study from Botswana,^22^ where researchers noted that RSV pneumonia in children induced more complications and longer duration of hospitalization, but mortality was lower in comparison to other agents. hMPV is recognized as a frequent etiological agent of pneumonia, causing severe disease.^23^ Here, we determined that detection of this virus was associated with hypoxemic pneumonia in children. These findings might be related to the fact that these two viruses in respiratory samples might be the etiological agents of pneumonia. Another hypothesis is that they might be associated with co-infections. However, we did not discern any relationship between infection type (bacterial, viral, or coinfection) and the risk of hypoxemia.

The main clinical predictors of hypoxemic pneumonia were lower chest indrawing and cyanosis with LR+ ratios between 2 and 3. If these signs are present, hypoxemia must be suspected in the absence of pulse oximetry, and oxygen therapy should be initiated promptly. Nevertheless, we did not discern that one sign had a high LR+ ratio, confirming that pulse oximetry is important for initial evaluation of pneumonia severity and should be implemented more widely in developing countries.^4^ Lower chest indrawing has been reported to be predictive of hypoxemia in children from Nigeria,^8^ while breathing rate ≥ 60 cycles/minute has been encountered with hypoxemia in children from Papua New Guinea.^24^ Other predictors are less known. The designation and validation of a simple, robust score predicting hypoxemia might be useful in resource-limited settings.

*Streptococcus pneumoniae* is recognized as the main etiological agent of severe pneumonia and death from pneumonia in children.^25^ The diagnosis of pneumococcus pneumonia is, however, difficult at the individual level, because of the low sensitivity of blood culture, particularly in case of previous exposure to antibiotics. In addition, the clinical meaning of pneumococcus detection in nasal samples by molecular testing is difficult to interpret because of the high prevalence of *S. pneumoniae* respiratory carriage in asymptomatic children.^26^ We did not observe associations between pneumococcus detection in nasal samples and disease severity, although pneumococcus-positive blood PCR was linked with greater mortality. Molecular *S. pneumoniae* detection by blood PCR is thus helpful in identifying bacterial pneumonia cases with the poorest prognosis who might need intensive therapies. Interpretation of molecular methods, such as PCR, to identify the etiology of pneumonia in children is, however, challenging. Respiratory viruses can be identified in asymptomatic children, and secondary bacterial infections in the lungs can easily be missed by these methods. In addition, identification of *S. pneumoniae* in blood by highly sensitive PCR may detect children with nasopharyngeal colonization only, which could lead to potential misclassification bias, particularly when using results of nasopharyngeal specimens to determine the etiology of pneumonia in children. We must be cautious with results interpretation. Moreover, PCT was the major biomarker associated with in-hospital death in our cohort. Several studies previously found an association between PCT and the risk of death or bacterial disease.^27^^–^^30^ However, such linkage has rarely been seen in pneumonia-infected children living in developing countries.

The main strength of the present study is the prospective data collection on a standardized form at different sites, with advanced molecular diagnosis in all cases, which reinforces internal validity. Its main limitations include paucity of information on exposures before hospital admission (i.e., breastfeeding, food intake, or vitamin supplementation). In addition, microbiological diagnosis of pneumonia is difficult because sensitive and specific tests are not routinely available in practice. Thus, based on respiratory samples, we were unable to differentiate colonization from infection, particularly by *S. pneumoniae*.^31^ However, analysis of the relationship between results from different samples and severity was contributive: we did not find associations between nasal colonization by pneumococcus and disease severity, hypoxemia, or death. Finally, selection bias might have occurred because of patient recruitment in hospital with inclusion of more severe cases or patients with easier access to care. However, we did not discern significant heterogeneity regarding SO_2_ of infants at admission and in-hospital mortality, which suggests that the results might be generalizable to different settings. We must acknowledge that 41% of the study population was enrolled in two sites from India, which might limit external validity. This proportion is, however, in accordance with estimates of global pneumonia incidence and related mortality: India might have accounted for almost 30% of the total number of severe pneumonia cases in children and 40% of the number of deaths worldwide.^25^

In conclusion, RSV and hMPV could be major causes of hypoxemia in children with severe pneumonia in developing countries, while *S. pneumoniae* detection by blood PCR is predictive of high risk of in-hospital mortality. Viral etiology might be considered in hypoxemic patients, whereas in very severe pneumonia, which can lead to death, *S. pneumoniae* may be implicated as the primary cause, even in the absence of hypoxemia at admission. Tachypnea and lower chest indrawing could be useful indicators of possible hypoxemia requiring oxygen therapy. Pulse oximetry should be included for better diagnosis in developing countries. Given these findings, preventive measures, such as increased vaccination coverage of children in developing countries, oxygen therapy of hypoxemic patients, and intensive cardiovascular support, even in non-hypoxemic patients, would reduce the burden of death by pneumonia in children.

## Supplementary Material

Supplemental Table and Figure.
