# Supplementary material for: Severity of Pneumonia in Under 5-Year-Old Children from Developing Countries: A Multicenter, Prospective, Observational Study
Source: Am J Trop Med Hyg. 2017 May 1;97(1):68–76. doi: 10.4269/ajtmh.16-0733 (PMC5508893; doi:10.4269/ajtmh.16-0733)
Supplement: Supplementary file 1 [file tpmd160733.SD1.pdf]

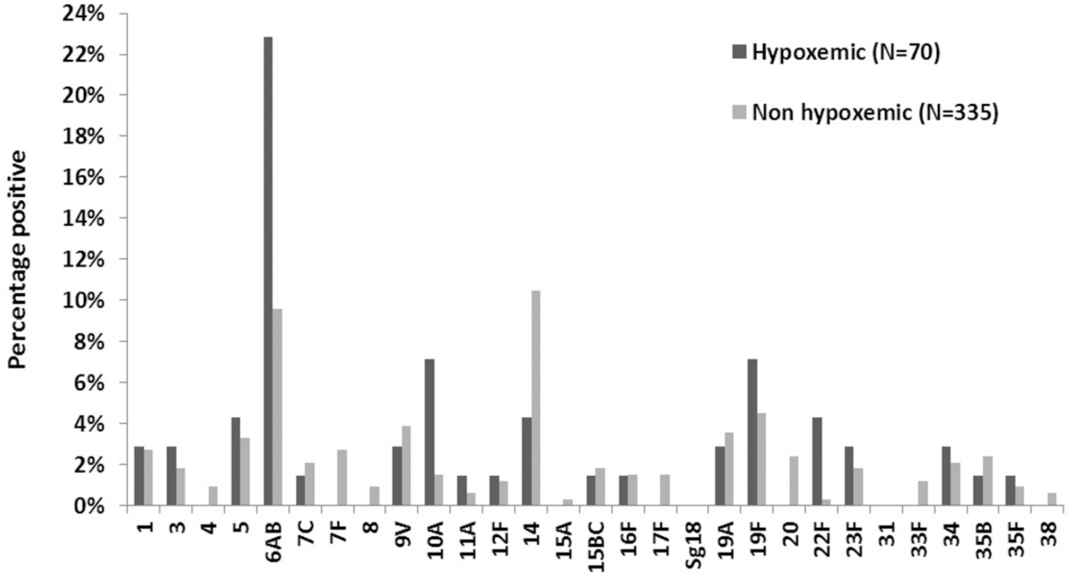


Pneumococcus serotypes 6AB and 10A were more frequent in hypoxemic compared to non-hypoxemic patients (22.9% vs. 9.5%, respectively, *P*=.002 and 7.1% vs. 1.5%, respectively, *P*=.006). The distributions of other serotypes were not significantly different in hypoxemic and non-hypoxemic children.

SUPPLEMENTAL FIGURE 1. Distribution of *Streptococcus pneumoniae* serotypes in hypoxemic and non-hypoxemic pneumonia children (*N* = 405).

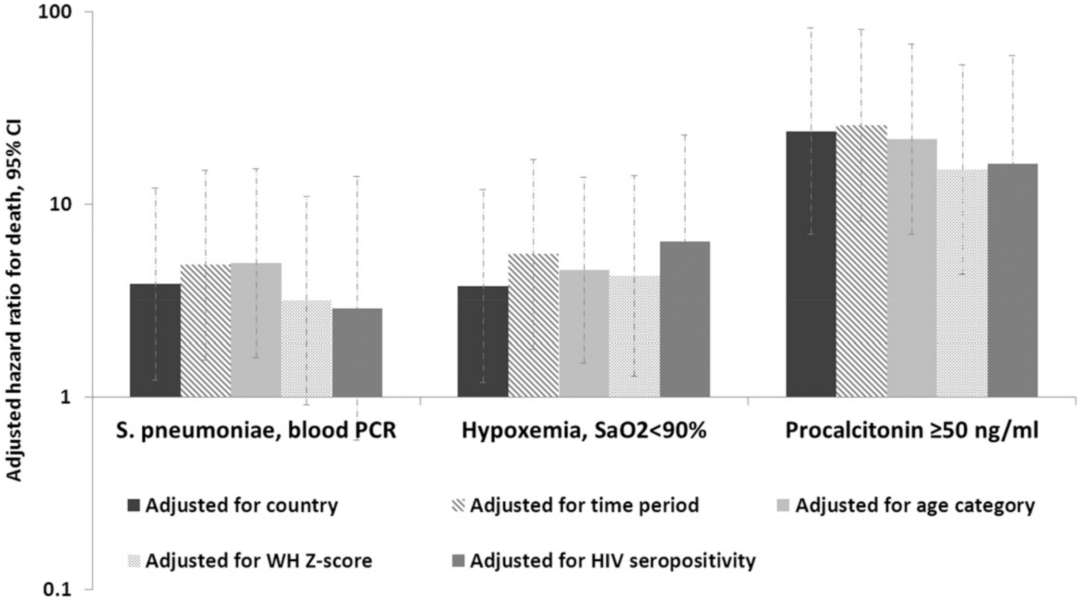


Among the 405 children with pneumonia, 13 died within 14 days after hospital admission, 392 were alive at discharge.
Adjusted hazards ratio for death was estimated by bivariate Cox proportional hazard model analysis, adjusted on country, patient age category (2-11 months, 12-23 months, 24-60 months), HIV seropositivity, time period per quarter and weight-for-height Z-score.

SUPPLEMENTAL FIGURE 2. Bivariate survival analysis of factors associated with in-hospital survival in children with pneumonia (*N* = 405).

Supplemental Table 1

Clinical and para-clinical signs and symptoms at admission associated with 2-week hospital death in children with pneumonia (*N* = 405)

| Characteristics at admission | Deceased (*N* = 13) | Alive (*N* = 392) | *P* |
|---|---|---|---|
| Demographic | | | |
| Gender, male, % (*N*) | 6/13 (46.1)* | 229/392 (58.4)* | 0.40 |
| Age, months, median (IQR) | 10 (7–18) | 15 (8–27.5) | 0.30 |
| Age category | | | 0.55 |
| 2–11 months | 7/13 (53.8) | 157/392 (40.0) | |
| 12–23 months | 3/13 (23.1) | 98/392 (25.0) | |
| 24–60 months | 3/13 (23.1) | 137/392 (34.9) | |
| Weight-for-height *Z* score ≤ 2 SD | 6/11 (54.5) | 86/289 (29.8) | 0.10 |
| Medical history | | | |
| Heart disease | 1/13 (7.7) | 26/391 (6.6) | 0.60 |
| Lung disease | 2/13 (15.4) | 34/389 (8.7) | 0.33 |
| Asthma | 0/13 (0) | 3/391 (0.8) | 0.99 |
| HIV positive | 1/10 (10.0) | 2/290 (0.7) | 0.10 |
| Contracted common cold/pharyngitis† | 1/13 (7.7) | 119/386 (30.8) | 0.12 |
| Contracted ILI† | 1/12 (8.3) | 27/375 (7.2) | 0.60 |
| Previous tuberculosis | 0/13 (0) | 2/390 (0.5) | 0.99 |
| Contact with a tuberculosis case | 0/11 (0) | 1/355 (0.3) | 0.99 |
| Prior treatment of fever | 7/13 (53.8) | 292/392 (75.5) | 0.11 |
| Pneumococcal conjugate vaccine | 0/12 (0) | 17/339 (5.0) | 0.99 |
| DPT-HepB-Hib vaccine, one dose | 8/12 (66.7) | 242/358 (67.6) | 0.99 |
| DPT-HepB-Hib vaccine, three doses | 5/11 (45.4) | 196/56.8) | 0.54 |
| Vital signs at admission | | | |
| Temperature, °C, median (IQR) | 38.2 (38–39.1) | 38.3 (37.7–38.8) | 0.83 |
| Breathing rate, cycles/minute, median (IQR) | 58 (56–70) | 56 (50–64) | 0.23 |
| Cardiac rate, cycles/minute, median (IQR) | 152 (140–158) | 140 (124–159) | 0.07 |
| Systolic pressure, mmHg, median (IQR) | 86 (87–100) | 86 (80–98) | 0.41 |
| Diastolic pressure, mmHg, median (IQR) | 60 (54–68) | 64 (60–70) | 0.40 |
| Clinical signs/symptoms at admission | | | |
| Dyspnea | 13/13 (100) | 378/392 (96.4) | 0.99 |
| Lower chest indrawing | 9/13 (69.2) | 311/390 (79.7) | 0.32 |
| Cough | 13/13 (100) | 384/390 (98.5) | 0.99 |
| Pulmonary crackles | 13/13 (100) | 350/391 (89.5) | 0.38 |
| Rhonchi | 1/10 (10.0) | 51/297 (17.2) | 0.99 |
| Wheezing | 3/13 (23.1) | 52/389 (13.4) | 0.40 |
| Rhinopharyngitis | 2/13 (15.4) | 70/390 (17.9) | 0.99 |
| Prostration or lethargy | 7/13 (53.8) | 131/391 (33.5) | 0.14 |
| Diarrhea | 1/13 (7.7) | 47/392 (12.0) | 0.99 |
| Cyanosis | 2/13 (15.4) | 24/390 (6.1) | 0.20 |
| Vomiting | 2/13 (15.4) | 54/390 (13.8) | 0.70 |
| Convulsions | 1/13 (7.7) | 15/391 (3.8) | 0.41 |
| Conjunctivitis | 0/13 (0) | 8/391 (2.0) | 0.99 |
| Diminished breath sounds | 4/10 (40.0) | 111/295 (37.6) | 0.99 |
| Dullness to percussion | 3/13 (23) | 99/389 (25.4) | 0.99 |
| Otitis | 0/13 (0)* | 3/391 (0.8)* | 0.99 |
| Rasping | 1/10 (10.0) | 45/297 (15.1) | 0.99 |
| Skin rash | 0/13 (0) | 12/392 (3.1) | 0.99 |
| Radiology | | | |
| Generalized dense homogenous opacification | 6/13 (46.1) | 116/390 (29.7) | 0.23 |
| Other findings | 7/13 (53.9) | 274/390 (70.3) | |
| Pleural effusion | 4/13 (30.8) | 79/388 (20.4) | 0.48 |
| Biology at admission | | | |
| White blood cell count, $\times 10^9$ cells/L, median (IQR) | 19.9 (5.2–25) | 12.5 (1–25.5) | 0.40 |
| Neutrophils, %, median (IQR) | 37 (32–53) | 46.8 (28–65) | 0.59 |
| C reactive protein, mg/L, median (IQR) | 48 (9–105) | 24 (6–90) | 0.42 |
| Antibiotic urinary test | 8/9 (88.9) | 217/243 (88.1) | 0.99 |

DPT-HepB-Hib = diphtheria, pertussis, tetanus, hepatitis B, and *Haemophilus influenzae* type b; ILI = influenza-like illness; IQR = interquartile range; SD = standard deviation; SO2 = oxygen saturation.

* Expressed as number/number from available data (%) unless otherwise specified.

† Within 2 weeks.

Supplemental Table 2

Microbiological agents associated with death within 2 weeks after hospital admission in children with pneumonia (*N* = 405)

| Microbiological agent | Deceased (*N* = 13) | Alive (*N* = 392) | *P* | Crude hazard ratio* (95% CI) |
|---|---|---|---|---|
| Nasal swab/aspirate | | | | |
| *Streptococcus pneumoniae* | 7/13 (53.8)† | 239/392 (61.0)† | 0.60 | 0.9 (0.3–2.6) |
| *Staphylococcus aureus* | 3/13 (23.1) | 67/391 (17.1) | 0.58 | 1.5 (0.4–5.3) |
| *Haemophilus influenzae* | 1/13 (7.7) | 20/392 (5.1) | 0.68 | 1.6 (0.2–12.2) |
| *Mycoplasma pneumoniae* | 0/13 (0) | 3/392 (0.8) | 0.75 | NE |
| *Chlamydia* spp. | 0/13 (0) | 1/392 (0.3) | 0.85 | NE |
| Human metapneumovirus | 2/13 (15.4) | 31/392 (7.9) | 0.33 | 2.4 (0.5–10.7) |
| Coronavirus 63 | 0/13 (0) | 2/392 (0.5) | 0.80 | NE |
| Coronavirus 229 | 0/13 (0) | 4/392 (1.0) | 0.71 | NE |
| Coronavirus 43 | 1/13 (7.7) | 14/392 (3.6) | 0.44 | 2.0 (0.3–15.7) |
| HKU | 1/13 (7.7) | 10/392 (2.5) | 0.26 | 4.2 (0.5–33.1) |
| Adenovirus | 1/13 (7.7) | 29/392 (7.4) | 0.97 | 1.2 (0.2–9.2) |
| Enterovirus | 0/13 (0) | 26/392 (6.6) | 0.34 | NE |
| Parechovirus | 0/13 (0) | 2/392 (0.5) | 0.80 | NE |
| Rhinovirus | 2/13 (15.4) | 112/392 (28.6) | 0.30 | 0.5 (0.1–2.1) |
| RSV | 0/13 (0) | 62/392 (15.8) | 0.12 | NE |
| hPIV 1 | 1/13 (7.1) | 15/392 (3.8) | 0.48 | 2 (0.3–15.2) |
| hPIV 3 | 1/13 (7.7) | 21/392 (5.4) | 0.71 | 1.4 (0.2–10.7) |
| hPIV 4 | 0/13 (0) | 12/392 (3.1) | 0.52 | NE |
| Influenza virus A | 2/13 (15.4) | 26/392 (6.6) | 0.22 | 2.2 (0.5–10.0) |
| Influenza virus B | 0/13 (0) | 8/392 (2.0) | 0.60 | NE |
| Influenza virus A H1/N1 | 1/13 (7.7) | 10/392 (7.7) | 0.26 | 3.1 (0.4–24.1) |
| Bocavirus | 0/13 (0) | 26/392 (6.6) | 0.34 | NE |
| Blood | | | | |
| *Staphylococcus aureus* | 0/13 (0) | 8/392 (2.0) | 0.60 | NE |
| *Haemophilus influenzae* | 1/13 (7.7) | 17/392 (4.3) | 0.56 | 2.2 (0.3–16.6) |

CI = confidence interval; hPIV = human parainfluenza virus; NE = non-estimable; RSV = respiratory syncytial virus.

* After univariate Cox regression analysis

† Expressed as number/number with available data (%) unless specified otherwise.
